# Edge responses are different in edges under natural versus anthropogenic influence: a meta‐analysis using ground beetles

**DOI:** 10.1002/ece3.2722

**Published:** 2017-01-18

**Authors:** Tibor Magura, Gábor L. Lövei, Béla Tóthmérész

**Affiliations:** ^1^ Department of EcologyUniversity of DebrecenDebrecenHungary; ^2^ MTA‐DE Biodiversity and Ecosystem Services Research GroupUniversity of DebrecenDebrecenHungary; ^3^ Department of AgroecologyFlakkebjerg Research CentreAarhus University, SlagelseDenmark

**Keywords:** anthropogenic edges, dispersal, edge effect, filter function, forest edges, forest species, invasion, matrix species, natural edges, species richness

## Abstract

Most edges are anthropogenic in origin, but are distinguishable by their maintaining processes (natural vs. continued anthropogenic interventions: forestry, agriculture, urbanization). We hypothesized that the dissimilar edge histories will be reflected in the diversity and assemblage composition of inhabitants. Testing this “history‐based edge effect” hypothesis, we evaluated published information on a common insect group, ground beetles (Coleoptera: Carabidae) in forest edges. A meta‐analysis showed that the diversity‐enhancing properties of edges significantly differed according to their history. Forest edges maintained by natural processes had significantly higher species richness than their interiors, while edges with continued anthropogenic influence did not. The filter function of edges was also essentially different depending on their history. For forest specialist species, edges maintained by natural processes were penetrable, allowing these species to move right through the edges, while edges still under anthropogenic interventions were impenetrable, preventing the dispersal of forest specialists out of the forest. For species inhabiting the surrounding matrix (open‐habitat and generalist species), edges created by forestry activities were penetrable, and such species also invaded the forest interior. However, natural forest edges constituted a barrier and prevented the invasion of matrix species into the forest interior. Preserving and protecting all edges maintained by natural processes, and preventing anthropogenic changes to their structure, composition, and characteristics are key factors to sustain biodiversity in forests. Moreover, the increasing presence of anthropogenic edges in a landscape is to be avoided, as they contribute to the loss of biodiversity. Simultaneously, edges under continued anthropogenic disturbance should be restored by increasing habitat heterogeneity.

## 1 Introduction

Worldwide fragmentation and loss of natural habitats increase the occurrence of habitat edges (also termed ecotones, boundaries, borders, and interfaces) that are transitional zones between adjoining ecosystems or habitats (Ewers & Didham, 2008; Lövei, Magura, Tóthmérész, & Ködöböcz, 2006; Ries, Fletcher, Battin, & Sisk, 2004). Abiotic conditions at habitat edges substantially differ from those in either adjacent habitats (Ewers & Didham, 2006; Murcia, 1995). This can have direct impacts on the spatio‐temporal distribution and dynamics of many species, as well as modify species interactions (predation, parasitism, competition, herbivory, pollination, and seed dispersal; Murcia, 1995). These abiotic, direct, and indirect biotic changes collectively constitute the so‐called edge effect (Murcia, 1995). Because of the importance and ubiquity of edges, ecological responses to their presence have been extensively researched (Ries et al., 2004). Ries and Sisk (2004) and Ries et al. (2004) developed a unified model predicting changes in abundance near edges for any species in any landscape. This unified model identifies ecological flows, access to spatially separated resources, resource mapping, and species interactions as fundamental mechanisms that change species abundance patterns across habitat edges. However, some variability remains unexplained, which makes generalizations difficult (Ries & Sisk, 2010). Edge orientation (Ries et al., 2004), temporal effects (Ries et al., 2004), habitat fragmentation effects (Hardt et al., 2013; Ries et al., 2004), edge contrast (Peyras, Vespa, Bellocq, & Zurita, 2013; Ries et al., 2004), magnitude of the edge effect (Ewers & Didham, 2006), species traits (Carvajal‐Cogollo & Urbina‐Cardona, 2015; Peyras et al., 2013), and habitat suitability (Peyras et al., 2013) were identified as possible factors responsible for the remaining variation.

However, habitat edges may also differ in their origin and maintaining processes; therefore the age, history, and the origin of edges can also be important drivers of the edge effect (Strayer, Power, Fagan, Pickett, & Belnap, 2003). The history of a habitat edge may determine its structural and functional properties and its ecological conditions (Strayer et al., 2003). In tropical forest patches, the function and structure of edges depend on their history, and the permeability of edges decreases with their development (“edge sealing”; Williams‐Linera, 1990). This can be generalized into an edge history hypothesis: Edges created by forces no longer in operation, and maintained only by natural processes (mainly by succession) and edges repeatedly disturbed by anthropogenic activities (forestry, agriculture, urbanization) have different structural and functional characteristics and have different influence on species richness and assemblage composition (Strayer et al., 2003; Turner, Gardner, & O'Neill, 2001).

Although the history and maintenance of habitat edges have relevance, their impact on the edge effect has not yet been tested. Here, we report the results of a meta‐analysis, synthesizing the effects of edges with different history on a common and widespread invertebrate group. We focused on forest edges, which are one of the most common habitat edges within terrestrial landscapes (Murcia, 1995). We distinguished forest edges maintained by natural processes (succession, irregular extensive grazing, and irregular mowing) from edges repeatedly disturbed and maintained by anthropogenic influence. Ground beetles (Coleoptera: Carabidae) were selected as study objects because they are taxonomically well known, common in most terrestrial habitats (Lövei & Sunderland, 1996), may serve as a keystone group (Mills, Soulé, & Doak, 1993), have often been used as indicators of environmental quality (Spellerberg, 1994), and there exist sufficient data on carabids in forest edges to make them suitable for testing the edge effect. Studies of edge effect on ground beetles reported findings that are contradictory or inconsistent. Some papers reported higher species richness in edges than in the forest interiors (e.g., Magura, 2002; Magura, Tóthmérész, & Molnár, 2001), while others showed no significant difference in species richness between edges and interiors (e.g., Kotze & Samways, 2001; Taboada, Kotze, & Salgado, 2004).

We hypothesized that the reported inconsistency is caused by differences between forest edges maintained by natural processes vs. those under repeated anthropogenic influence (e.g., forestry, agriculture, urbanization). More specifically, we hypothesized that forest edges maintained by natural processes have significantly higher carabid diversity than their interiors, while edges with continued anthropogenic influence do not. We also hypothesized that species with different habitat affinities show idiosyncratic responses to forest edges, and edges still under anthropogenic influences are more easily penetrable for matrix species. We call this the “history‐based edge effect” hypothesis. We tested these hypotheses by a meta‐analysis using subgroup analyses, which is an appropriate method to determine whether the edge effect on ground beetles differs significantly at edges with different history (Borenstein, Hedges, Higgins, & Rothstein, 2009).

Our analysis shows that carabid diversity is higher only at edges not under continued human influence. We also found that the edges are penetrable for certain groups of species while impermeable to others, depending on the origin and development of the edges, supporting the history‐based edge effect hypothesis.

## 2 Materials and Methods

### 2.1 Literature search and data selection

We collected data by performing a literature search on Web of Science (now incorporating several biological databases) for the period 1975–2015, using the following search terms: TOPIC = (forest* OR woodland*) AND TOPIC = (edge* OR margin* OR ecotone*) AND TOPIC = (carabid* OR ground beetle*). Additionally, we scanned the reference section of the publications found in this search for additional, undetected, relevant publications. We did not distinguish by publication forum, and “gray literature” was also considered to avoid selection bias (Pullin & Stewart, 2006). To be included, a paper had to report data on carabid abundance and/or species richness, comparing at least a clearly defined forest interior and a forest edge. Data were extracted from text, tables, and graphs. From papers that studied carabids along transects, only data from the interiormost forest locations were used. Distance of the innermost forest locations from the edges was 25–150 m; thus, samples from the forest locations could be regarded as statistically independent from those in the edges (Digweed, Currie, Cárcamo, & Spence, 1995). Samples in the forest edges were collected in the 0–15 m edge zone, where 0 m represents the line of outermost trees.

### 2.2 Classification of edges based on their origin

Forest edges were classified according to their maintaining processes. Edges whose neighboring habitats have been unmanaged (forest interiors without fire, cutting or thinning; adjacent grasslands or meadows without burning, intensive grazing, or mowing) for at least 50 years were classified as edges maintained by natural processes. These edges are maintained by natural processes (such as succession) or irregular interventions (irregular mowing and irregular extensive grazing), with succession starting between such disturbance events. Edges created by forestry activities (clear‐cutting, forest management), urbanization (forest patches embedded in, and adjacent to an urbanized area) or agriculture (the neighboring habitat cultivated or intensively grazed, mowed, and/or regularly burned), and repeatedly disturbed by such operations were termed edges under continued anthropogenic influence. Anthropogenic influences, including forestry operations, management of the urban environment, tillage, pesticide, herbicide and fertilizer use, intensive grazing, mowing, and repeated fires lead to simplified forest edges (Boutin & Jobin, 1998; Harper et al., 2005), because these disturbances repeatedly disrupt population, community, and ecosystem structures, and change resource availability, substrate structure, and/or the physical environment (Pickett & White, 1985). Forest edges maintained by natural processes are well structured, have stratified vegetation layers, and contain a mixture of plant species from the adjoining habitats (Forman & Godron, 1986).

### 2.3 Evaluation methods

At the assemblage level, the mean overall abundance and species richness of ground beetles (Coleoptera: Carabidae) were analyzed. Species with different habitat affinities may show different responses to edge effect; therefore, ground beetles were classified by habitat preference, distinguishing (1) forest specialists (species associated with forest habitats); (2) habitat generalists (species occurring both in forests and other habitats); and (3) species associated with open habitats. Such information was accepted if stated in the original paper; if not given, it was retrieved from the literature (Bousquet, 2010; Freude, Harde, & Lohse, 1989; Hůrka, 1996; Lindroth, 1985), or from Internet databases. Species whose habitat affinity could not be unequivocally categorized were not included into the analyses. Subsequently, forest, generalist, and open‐habitat species were evaluated separately.

### 2.4 Statistical analyses

For each edge‐to‐interior comparison, a common effect size, the unbiased standardized mean difference (Hedges’ *g*) was calculated between forest interior and forest edge:

(1) $$g=J\frac{\bar{X_{F}}-\bar{X_{E}}}{S_{\mathrm{within}}}$$

(2) $$S_{\mathrm{within}}=\sqrt{\frac{\left(n_{F}-1\right)S_{F}^{2}+\left(n_{E}-1\right)S_{E}^{2}}{n_{F}+n_{E}-2}}$$

and

(3) $$J=1-\frac{3}{4(n_{F}+n_{E}-2)-1},$$

where $\bar{X_{F}}$ and $\bar{X_{E}}$ are the mean abundance or species richness of forest interior and forest edge, respectively, *n*_F_ and *n*_E_ are the sample sizes of the forest interior and forest edge, and *S*_F_ and *S*_E_ are their respective SD values. A negative *g* value means higher abundance or species richness in forest edges than interiors.

We used subgroup meta‐analysis to determine whether the edge has an effect on ground beetle abundance and species richness according to forest edge history. The two main groups were forest edges maintained by natural or anthropogenic processes. Edges with anthropogenic disturbances were further divided into subgroups based on the type of human influence (forestry, urbanization, or agriculture). We estimated the overall effect and examined the effects of moderators (edge history; type of anthropogenic influence) using a random‐effects model. The random‐effects model was used because studies were not expected to estimate a common effect size due to variation in regions, locations, conditions, experimental setups, and research methods used in the individual studies (Borenstein et al., 2009). Random‐effect models are more plausible than fixed‐effect ones because they attribute the distribution of effect sizes to real differences among studies and do not assume sampling error as the only source of differences (Borenstein et al., 2009). The mean effect size was considered statistically significant if the 95% bootstrap confidence interval (CI; calculated with 999 iterations) did not include zero.

We assessed whether effect sizes were homogenous or varied across studies (i.e., if there was heterogeneity), because if effect sizes vary across studies, the interpretation of results will be substantially different than under consistent effect sizes. To describe the heterogeneity, complementary measures, *Q*,* T* ^*2*^, and *I* ^*2*^, were estimated (Borenstein et al., 2009). Using a *Q*‐test based on analysis of variance, we partitioned the total variance (*Q*_total_) into within‐ (*Q*_within_) and between group (*Q*_between_) variances, and these were tested for statistical significance (Borenstein et al., 2009). Significant variance between groups (*Q*_between_) means that edge effect on species richness or abundance significantly differed according to the history or the continued anthropogenic influence. To evaluate the proportion of true variance explained by the covariates (subgroup classification), the *R* ^*2*^ was calculated (Borenstein et al., 2009). During the calculations, subgroups with less than five cases were excluded from subgroup (categorical) analyses.

Meta‐analyses are often exposed to publication bias resulting in missing studies and a potentially biased effect sizes (Borenstein et al., 2009). Therefore, we tested the publication bias using funnel plots and the Egger test (Borenstein et al., 2009). In case of significant asymmetry, the trim and fill method was used as suggested by Duval and Tweedie (2000). This method calculates the number of missing studies and estimates their effect sizes as well as standard errors; then, these missing studies are added to the data set and the summary effect size is recomputed. This method yields an unbiased estimate of the summary effect size (Borenstein et al., 2009). Meta‐analyses, heterogeneity measures, and assessing publication bias were completed by the *MAd* and *metafor* packages (Del Re & Hoyt, 2014; Viechtbauer, 2010) operated in the R version 3.2.0.

## 3 Results

The literature search yielded 204 publications. After applying the selection criteria, 53 papers were retained. Of these, mean abundance and/or species richness with standard deviations, and sample sizes for forest interiors and edges were recoverable from 39 publications. Twelve papers studied forest edges maintained by natural processes, 26 papers investigated edges maintained by continued anthropogenic interventions, and a single study examined both. Edges maintained by human influence were further grouped according to the activity type: forestry (10 papers), urbanization (three papers), or agriculture (13 papers). Studies were carried out on all continents (except Antarctica), with a majority from Europe (21 papers); the number of experiments from Asia (6) and North America (7) were almost equal. Few papers reported work on African, Australian (two each), and South American (1) forest edges (see Appendix S1).

### 3.1 Edge responses at assemblage level

**Figure 1:**
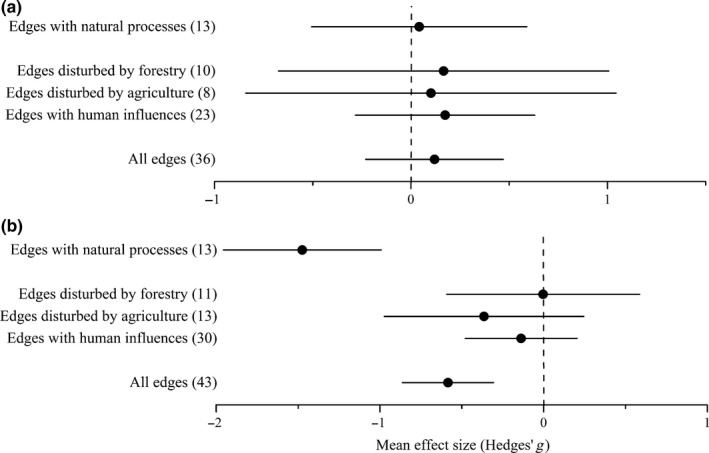
Mean effect sizes of random‐effect models (mean Hedges’ *g* ±95% confidence interval) for abundance (a) and species richness (b) of ground beetles. Values in brackets refer to the number of comparisons from which the mean effect size was calculated. A negative *g* value means higher abundance or species richness in forest edges than interiors. The mean effect size was considered statistically significant if the 95% bootstrap confidence interval (CI) did not include zero. “Edges with human influences” represents data from edges under anthropogenic influence (agriculture, forestry, industry, recreation, or urbanization)

Ground beetle abundance was not significantly different according to the history of edges (*Q*_between_=0.131, *df *= 1, *p *= .717; see Appendix S2): There was no significant difference in ground beetle abundance between forest edges and respective interiors, neither when edges were maintained by natural processes nor when edges had continued anthropogenic influence (Figure 1a). Total heterogeneity in the overall model, however, was significant (*Q*_total_ = 195.556, *df *= 35, *p *< .001; see Appendix S2), and there was also significant residual, unexplained heterogeneity (*Q*_within_ = 195.467, *df *= 34, *p *< .001; see Appendix S2). In anthropogenically maintained edges, the edge effect on abundance was not significantly related to the disturbance type (agriculture vs. forestry; *Q*_between_ = 0.010, *df *= 1, *p *= .920; see Appendix S2). The abundance of ground beetles in edges and their interiors was not significantly different when edges were under agricultural or forestry disturbance (Figure 1a). Both the total and the unexplained heterogeneities were significant (*Q*_total_ = 88.458, *df *= 17, *p *< .001 and *Q*_within_ = 88.347, *df *= 16, *p *< .001, respectively; see Appendix S2). Neither the classical nor the random‐effects version of the Egger test revealed significant asymmetry in the funnel plot, indicating the absence of publication bias (see Appendix S3).

Edge effect on species richness was significantly different according to edge history (*Q*_between_ = 19.636, *df *= 1, *p *< .001; see Appendix S2). Forest edges maintained by natural processes had significantly higher species richness than their interiors, while edges under continued anthropogenic influence showed no such difference (Figure 1b). Although the covariates accounted for substantial proportion of true variance (*R* ^*2*^ = 37.16%), there was still significant unexplained heterogeneity (*Q*_within_ = 159.651, *df *= 41, *p *< .001; see Appendix S2). The edge effect on species richness was not significantly related to the type of disturbance (*Q*_between_ = 0.696, *df *= 1, *p *= .404; see Appendix S2). In either type of anthropogenically disturbed edges (forestry or agriculture), the species richness in edges vs. interiors was not significantly different (Figure 1b). Both the total and the unexplained heterogeneities were significant (*Q*_total_ = 88.448, *df *= 23, *p *< .001 and *Q*_within_ = 88.291, *df *= 22, *p *< .001, respectively; see Appendix S2). There was no publication bias regarding species richness (see Appendix S3).

### 3.2 Edge responses by habitat affinity

**Figure 2:**
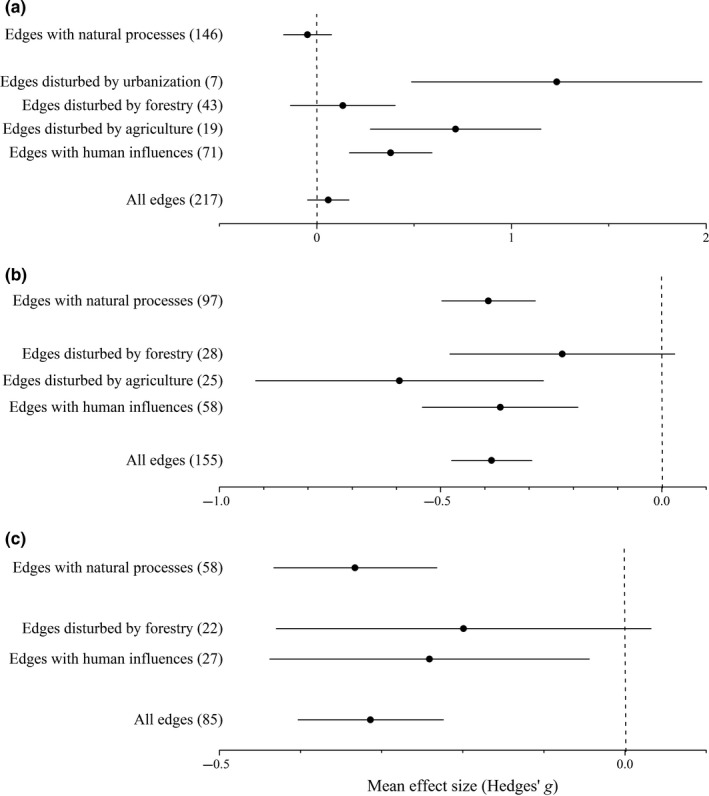
Mean effect sizes of random‐effect models (mean Hedges’ *g* ±95% confidence interval) for the abundance of forest specialist (a), generalist (b), and open‐habitat ground beetle species (c). Values in brackets refer to the number of species for whose abundance the mean effect size was calculated. A negative *g* value means higher abundance in forest edges than interiors. The mean effect size was considered statistically significant if the 95% bootstrap confidence interval (CI) did not include zero. “Edges with human influences” represents data from edges under anthropogenic influences (agriculture, forestry, industry, recreation, or urbanization)

The edge effect on the abundance of forest specialist species was related to edge history (*Q*_between_ = 11.733, *df *= 1, *p *= .001; see Appendix S2). The abundance of forest species was not significantly different between edges maintained by natural processes and their interiors, but was significantly lower in the edges than in the interior in the case of edges under anthropogenic disturbance (Figure 2a). The classification of edges according to the maintaining processes (covariates) accounted for a small proportion only of the true variance (*R* ^*2*^ = 4.14%); consequently, both the total and the unexplained heterogeneities were significant (*Q*_total_ = 1008.142, *df *= 216, *p *< .001 and *Q*_within_ = 983.506, *df *= 215, *p *< .001, respectively; see Appendix S2). Nonetheless, the edge effect on the abundance of forest species significantly differed according to the type of human disturbance (*Q*_between_ = 10.439, *df *= 2, *p *= .005; see Appendix S2). In edges disturbed by agriculture or urbanization, significantly fewer forest specialists were in the edges than their interiors, while there was no such significant difference in forestry‐influenced edges (Figure 2a). Both the total and the unexplained heterogeneities were significant (*Q*_total_ = 185.32, *df *= 68, *p *< .001 and *Q*_within_ = 168.443, *df *= 66, *p *< .001, respectively; see Appendix S2). Both regression tests showed significant funnel plot asymmetries. The trim and fill method estimated 15 missing values, but adding these did not change the nonsignificance of the overall effect in the model (see Appendix S3).

The edge effect on the abundance of generalist species was not significantly different by either the history of edges or the type of human influence (*Q*_between_ = 0.064, *df *= 1, *p *= .8 and *Q*_between_ = 3.061, *df *= 1, *p *= .08, respectively; see Appendix S2). Overall, the abundance of generalist species was significantly higher in the edges than interiors. However, abundance was not significantly different between edges disturbed by forestry and their respective interiors (Figure 2b). In all models, both the total and the unexplained heterogeneities were significant (see Appendix S2). The random‐effects version of the Egger test indicated funnel plot asymmetry, but the trim and fill procedure (adding 38 data points) did not change the significance of the overall effect (see Appendix S3).

The edge effect on the abundance of open‐habitat species was not related to the edge history (*Q*_between_ = 0.664, *df *= 1, *p *= .415; see Appendix S2). Their abundance was always higher in the edges than interiors, both in edges maintained by natural processes and anthropogenically maintained edges. However, forestry‐influenced edges and their interiors did not have significantly different numbers of open‐habitat individuals (Figure 2c). Neither the total nor the unexplained heterogeneities were significant (see Appendix S2). There was asymmetry in the funnel plot, but the recomputed model did not give a different outcome, even though 23 missing values were added (see Appendix S3).

## 4 Discussion

### 4.1 Diversity‐enhancing properties of the edges

Forest edges maintained by natural processes have a stratified horizontal structure, with a shrub and sapling zone (so‐called mantel) toward the forest interior, and a perennial herb layer (saum) toward the open habitat (Forman & Godron, 1986). Because of this physiognomy, edges maintained by natural processes have a distinct microclimate, high habitat heterogeneity, and environmental conditions that vary at a modest amplitude (Cadenasso, Pickett, Weathers, & Jones, 2003; Harper et al., 2005). In contrast, forest edges still under anthropogenic influence are repeatedly exposed to direct (by forest management, management of urban environments, tillage, plowing, intensive grazing, mowing, and fires), and/or indirect (pesticide, herbicide, and fertilizer drift) disturbance. Therefore, edges under human influence have more widely fluctuating microclimatic and environmental conditions. Due to the dissimilar structure, environmental conditions, and habitat heterogeneity of edges of different history, their diversity‐enhancing properties are also different. Forest edges maintained by natural processes commonly contain species from both adjoining habitats but also species characteristic of, and often restricted to, the edge (Lacasella et al., 2015; Magura, 2002; Magura et al., 2001). Moreover, forest edges maintained by natural processes are rich in microhabitats and food supply (Cadenasso et al., 2003); therefore, many species visit these edges for feeding, reproducing, resting, and overwintering during their life cycles resulting in a higher species richness in these forest edges than in the adjoining habitats (Odum, 1971). Changes in vegetation structure and composition, microclimate, and microhabitats in the forest edges under human influence are detrimental for both species from the neighboring habitats and the edge‐preferring ones (Murcia, 1995). Consequently, overall species richness in such edges may not be higher than in the adjoining habitats as our analysis of data on ground beetles also testified. It seems likely that a similar pattern exists regarding spiders: higher spider species richness was reported in forest edges maintained by natural processes than their interiors (Horváth, Magura, Péter, & Tóthmérész, 2002; Lacasella et al., 2015), but not in the case of continued anthropogenic influence (Fuller, Irwin, Kelly, O'Halloran, & Oxbrough, 2013; Rodrigues, Mendonça, & Costa‐Schmidt, 2014).

### 4.2 Filter function of the edges

Forest edges are not only frequent structural components of the landscape; they also have important functions regulating biological processes, like dispersal or invasibility (Ries et al., 2004). Edges created and maintained by forestry (clear‐cutting, group felling, other forest management) seem to be permeable by matrix species (Strayer et al., 2003), as these edges allow the open‐habitat specialists and generalist species from the surrounding, non‐forested matrix to colonize the forest interior and the forest specialist species to move the other way. Invasion by open‐habitat and generalist species into the forest interior may cause decline or local extinction of native forest interior specialists and facilitate or accelerate further invasion by alien species, causing further habitat deterioration of both the edge (Pryke & Samways, 2012), and the interior (Pinheiro, Duarte, Diehl, & Hartz, 2010; Pryke & Samways, 2012). Such damaging effects on the species diversity, structure, and function of the forest interiors are increasing (Harper et al., 2005, 2015; Murcia, 1995). Contrary to this trend, open‐habitat and generalist species were significantly more abundant in forest edges maintained by natural processes, indicating an immigration pressure, but they were significantly less numerous in the forest interiors, suggesting that these edges operate as an impermeable filter, inhibiting these species to penetrate into the forest interior, and returning these species to the matrix habitat from which they originated (Strayer et al., 2003) or redirecting them to move along the edges (Wood & Samways, 1991). Therefore, forest edges maintained by natural processes mount biotic resistance against the invasion of matrix species into the forest interior. Biotic resistance can be caused by the presence of predators, competitors, lack of suitable food, nonpreferred fluctuations in habitat conditions, unsuitable egg‐laying sites, all of which may cause, alone or in concert, the habitat becoming a population sink for these species.

The abundance of forest specialist species showed no significant difference between the forest edges maintained by natural processes and their interiors, indicating that naturally maintained edges are permeable and suitable habitats for forest species. Although we have no direct proof, it is likely that they will have opportunities to disperse and may reach other forest fragments. Contrary to this, the abundance of forest specialist species was significantly lower in edges under continued anthropogenic influence than in the interiors meaning that such edges are practically impenetrable, discouraging the dispersal of forest specialists between forest fragments, thereby contributing to higher isolation in fragmented landscapes.

Our finding that the edge effect can be mediated by edge history is based on a single invertebrate group, ground beetles, which are at the consumer trophic level of the food web. Such influence may plausibly exist for other organisms with a different trophic position, mobility, development type, life history, or life span (see for butterflies Pryke & Samways, 2001, 2003). Cadenasso and Pickett (2001) showed that more seeds from the surrounding open landscape crossed through experimentally thinned forest edges than through the intact edge and also dispersed farther into the forest interior. Moreover, similarly to our results, previous studies on invertebrates also showed that species from the forest interior can move through the forest edges maintained by natural processes (for spider, centipedes, and ground beetles: Lacasella et al., 2015; for spiders: Gallé & Torma, 2009), while these natural forest edges prevent the matrix (open‐habitat) species to cross them (Lacasella et al., 2015). Forest remnants with anthropogenic edges, however, are invaded by species from the surrounding matrix (geometrid moths: Axmacher et al., 2004; ground and rove beetles: Knapp et al., 2013; spiders and ground beetles: Matveinen‐Huju, Koivula, Niemelä, & Rauha, 2009), but are repulsive for forest specialists (ground and rove beetles: Knapp et al., 2013; bark beetles: Peltonen & Heliövaara, 1998). These results also strengthen our hypothesis which predicts different filter function of edges depending on their history.

Abruptness, the rate at which forest transitions to the adjacent non‐forested habitat could be the main cause of the different filter function of forest edges with different histories (Bowersox & Brown, 2001). This usually has a spatial dimension: abrupt edges are often also narrower. Forest edges maintained by natural processes have a stratified structure and are highly heterogeneous, therefore display gradual changes in habitat structure and environmental conditions. The gradually changing structure and environmental conditions, on the one hand, permit the dispersion of forest specialist species from the forest interior to the edge. On the other hand, these gradual forest edges have a buffering capacity and prevent the neighboring open habitats to extend their “condition halo” into the forest interior, therefore inhibiting the invasion of generalist and open‐habitat species into the forest interior. Contrary to this, in edges under anthropogenic influences with simplified structure, the changes in habitat structure and environmental conditions are abrupt. These abrupt edges create unfavorable habitats for forest specialists, decreasing their dispersal from the forest interior into the edges and outside. Moreover, the buffering capacity of abrupt edges is limited; therefore, the environmental conditions (e.g., temperature, moisture) in forest interiors bordered by abrupt edges could allow the invasion of generalist and open‐habitat species into the forest interior.

### 4.3 Conservation, future directions, and challenges

Although the covariates (classification of edges according to their history or the type of human disturbance) accounted for a considerable proportion of the true variance, in all models there remained significant unexplained heterogeneity. The variance explained by the covariates (edge classification based on history) was also low, indicating the existence of other factors influencing the edge effect. This heterogeneity could arise from differences in biogeographical regions, and/or study designs and methods applied in the individual studies. Besides those features of edges identified in our study (history or maintaining processes, disturbance types), other inherent features of edges (e.g., size, isolation, type and quality of adjacent habitats, orientation, temporal effects, edge contrast; see Ewers & Didham, 2006; Ries et al., 2004) could also contribute to heterogeneity. Furthermore, each species (yet with the same habitat affinity) responds to edges in its own particular way, resulting a species‐dependent filtration by edges (Ingham & Samways, 1996). Therefore, all of the above mentioned features must be considered in future edge effect studies.

From the point of conservation, preserving and protecting all edges maintained by natural processes, and preventing unfavorable changes to their structure, composition, and characteristics will better protect the quality of forest interiors. Anthropogenic edges continue to be frequently created, for example, by forestry practices in boreal forests in Canada and Fennoscandia (Harper et al., 2015). The remaining intact forest patches after anthropogenic interventions are important source habitats. The role of patch size, configuration, distance between patches, corridors, and the nature of the matrix are instrumental to insure dispersal between the fragments, particularly for forest specialist species (Naaf & Kolk, 2015). Our data now add support to the idea that boundaries, like forest edges, are also important in conservation management (Samways, 2007). Forest edges under continued anthropogenic influence can hamper the dispersal of forest specialists between habitat patches, obstructing metapopulation processes (Jordán, Magura, Tóthmérész, Vasas, & Ködöböcz, 2007). Therefore, the increasing presence of anthropogenic edges in a landscape is to be avoided, as they contribute to forest degradation and the loss of biodiversity (Harper et al., 2005). Simultaneously, the restoration of edges under continued anthropogenic intervention is an urgent task in conservation management. Promoting habitat heterogeneity and reducing the contrast between these edges and the surrounding habitats (softening the edges) to encourage movement of forest specialist species through the edges are crucial tasks during restoration (Anderson & Carter, 1987; Samways, 2007).

## Data Accessibility

Publications used in the meta‐analyses are uploaded as online supporting information.

## Conflict of Interest

None declared.
