# Supplementary material for: Edge responses are different in edges under natural versus anthropogenic influence: a meta‐analysis using ground beetles
Source: Ecol Evol. 2017 Jan 18;7(3):1009–17. doi: 10.1002/ece3.2722 (PMC5288263; doi:10.1002/ece3.2722)
Supplement: Supplementary file 1 [file ECE3-7-1009-s001.docx]

**SUPPORTING INFORMATION**

Magura T, Lövei GL, and Tóthmérész B.

Edge responses are different in edges under natural versus anthropogenic influence: a meta-analysis using ground beetles. Ecol Evol. doi:10.1002/ece3.2722.

**Appendix S1.** Publications used in the meta-analyses, which reported mean values of abundances and/or species richness, standard deviation, and sample size for both the forest interior and the forest edge.

| **Maintaining forces**  **of the edge** | **Continent** | **Country** | **Number of sampled** | | **Reference** |
| --- | --- | --- | --- | --- | --- |
|  |  |  | **individuals** | **species** |  |
| Anthropogenic |  |  |  |  |  |
| agriculture | Africa | South Africa | 984 | 30 | Kotze & Samways 1999 |
| agriculture | Africa | South Africa | 4042 | 23 | Kotze & Samways 2001 |
| agriculture | Asia | Japan | 2235 | 1^*^ | Kagawa & Maeto 2009 |
| agriculture | Asia | Japan | 1268 | 36 | Kagawa & Maeto 2014 |
| agriculture | Asia | China | 2343 | 33 | Yu et al. 2007 |
| agriculture | Australasia | New Zealand | 71 | 2^**^ | Ewers 2008 |
| agriculture | Europe | UK | 12755 | 50 | Bedford & Usher 1994 |
| agriculture | Europe | Finland | 3709 | 64 | Halme & Niemelä 1993 |
| agriculture | Europe | Switzerland | 853 | 33 | Hänggi & Baur 1998 |
| agriculture | Europe | France | 2014 | 48 | Roume et al. 2011b |
| agriculture | Europe | Poland | 774 | 6^***^ | Sklodowski 1999 |
| agriculture | Europe | Spain | 5436 | 43 | Taboada et al. 2004 |
| agriculture | South America | Brazil | 577 | 26 | Cividanes et al. 2010 |
| forestry | Asia | China | 27986 | 25 | Yu et al. 2009 |
| forestry | Asia | China | 402 | 19 | Yu et al. 2010 |
| forestry | Australasia | Australia | no data | 45 | Davies & Margules 1998 |
| forestry | Europe | Belgium | 1221 | 1^*^ | Gaublomme et al. 2013 |
| forestry | Europe | Finland | 5609 | 34 | Heliölä et al. 2001 |
| forestry | North America | USA | 12413 | 31 | Halaj et al. 2008 |
| forestry | North America | Canada | 15799 | 28 | Lemieux & Lindgren 2004 |
| forestry | North America | Canada | 2494 | 35 | Phillips et al. 2006 |
| forestry | North America | Canada | 1924 | >35^****^ | Spence et al. 1996 |
| forestry | North America | USA | 5498 | 87 | Ulyshen et al. 2006 |
| industry | North America | USA | 1254 | 59 | Silverman et al. 2008 |
| recreation | North America | USA | 933 | 37 | Strong et al. 2002 |
| urbanization | Asia | Japan | 1503 | 23 | Soga et al. 2013 |
| urbanization | Europe | Belgium | 53594 | 100 | Gaublomme et al. 2008 |
| urbanization | Europe | Belgium | 1221 | 1^*^ | Gaublomme et al. 2013 |
| Natural |  |  |  |  |  |
| natural | Europe | Croatia | 20526 | 66 | Brigić et al. 2014 |
| natural | Europe | Hungary | 4696 | 31 | Elek & Tóthmérész 2010 |
| natural | Europe | Switzerland | 853 | 33 | Hänggi & Baur 1998 |
| natural | Europe | Italy | 3211 | 44 | Lacasella et al. 2015 |
| natural | Europe | Hungary | 1373 | 20 | Magura & Tóthmérész 1997 |
| natural | Europe | Hungary | 2221 | 22 | Magura & Tóthmérész 1998 |
| natural | Europe | Hungary | 1714 | 32 | Magura 2002 |
| natural | Europe | Hungary | 4339 | 40 | Magura et al. 2000 |
| natural | Europe | Hungary | 3406 | 34 | Magura et al. 2001 |
| natural | Europe | Hungary | 3009 | 22 | Magura et al. 2002 |
| natural | Europe | Romania | 5662 | 53 | Máthé 2006 |
| natural | Europe | Hungary | 1633 | 26 | Molnár et al. 2001 |
| natural | Europe | Hungary | 3006 | 57 | Tóthmérész et al. 2014 |

^*^ Only one ground beetle species was studied

^**^ Only two ground beetle species were studied

^***^ Only six ground beetle species were studied

^****^ The exact number of sampled species was not reported

***References***

Bedford, S.E. & Usher, M.B. (1994) Distribution of arthropod species across the margins of farm woodlands. *Agriculture, Ecosystems & Environment*, 48, 295−305.

Brigić, A.,Starčević, M., Hrašovec, B. & Elek, Z. (2014) Old forest edges may promote the distribution of forest species in carabid assemblages (Coleoptera: Carabidae) in Croatian forests. *European Journal of Entomology*, 111, 715−725.

Cividanes, F.J., Araújo, E.S., Ide, S. & Galli, J.C. (2010) Distribution and habitat preference of Carabidae andStaphylinidae (Coleoptera) in an orange orchard and a forest fragment. *Florida Entomologist*, 93, 339−345.

Davies, K.F. & Margules, C.R. (1998) Effects of habitat fragmentation on carabid beetles: experimental evidence. *Journal of Animal Ecology*, 67, 460−471.

Elek, Z. & Tóthmérész, B. (2010) Carabid beetles among grassland-forest edge-beech forest habitats in Northern Hungary. *Community Ecology*, 11, 211−216.

Ewers, R.M. (2008) Spatio-temporal variation in mortality rates of *Mecodema* spp. (Coleoptera: Carabidae) across a forest-grassland edge in New Zealand. *Insect Conservation and Diversity*, 1, 40−47.

Gaublomme, E., Hendrickx, F., Dhuyvetter, H. & Desender, K. (2008) The effects of forest patch size and matrix type on changes in carabid beetle assemblages in an urbanized landscape. *Biological Conservation*, 141, 2585−2596.

Gaublomme, E., Maebe, K., Van Doninck, K., Dhuyvetter, H., Li, X., Desender, K. & Hendrickx, F. (2013) Loss of genetic diversity and increased genetic structuring in response to forest area reduction in a ground dwelling insect: a case study of the flightless carabid beetle *Carabus problematicus* (Coleoptera, Carabidae). *Insect Conservation and Diversity*, 6, 473−482.

Halaj, J.,Halpern, C.B. & Yi, H. (2008) Responses of litter-dwelling spiders and carabid beetles to varying levels and patterns of green−tree retention. *Forest Ecology and Management*, 255, 887−900.

Halme, E. & Niemelä, J. (1993) Carabid beetles in fragments of coniferous forest. *Annales Zoologici Fennici*, 30, 17–30.

Hänggi, A. & Baur, B. (1998) The effect of forest edge on ground-living arthropods in a remnant of unfertilized calcareous grassland in the Swiss Jura mountains. *Mitteilungen der Schweizerischen Entomologischen Gesellschaft*, 71, 343−354.

Heliölä, J., Koivula, M. & Niemelä, J. (2001) Distribution of carabid beetles (Coleoptera, Carabidae) across a boreal forest-clearcut ecotone. *Conservation Biology*, 15, 370−377.

Kagawa, Y. & Maeto, K. (2009) Spatial population structure of the predatory ground beetle *Carabus yaconinus* (Coleoptera: Carabidae) in the mixed farmland-woodland satoyama landscape of Japan. *European Journal of Entomology*, 106, 385−391.

Kagawa, Y. & Maeto, K. (2014) Ground beetle (Coleoptera: Carabidae) assemblages associated with a satoyama landscape in Japan: the effects of soil moisture, weed height, and distance from woodlands. *Applied Entomology and Zoology*, 49, 429−436.

Kotze, D.J. & Samways, M.J. (1999) Invertebrate conservation at the interface between the grassland matrix and natural Afromontane forest fragments. *Biodiversity and Conservation*, 8, 1339−1363.

Kotze, D.J. & Samways, M.J. (2001) No general edge effects for invertebrates at Afromontane forest/grassland ecotones. *Biodiversity and Conservation*, 10, 443−466.

Lacasella, F., Gratton, C., De Felici, S., Isaia, M., Zapparoli, M., Marta, S. & Sbordoni, V. (2015) Asymmetrical responses of forest and "beyond edge" arthropod communities across a forest-grassland ecotone. *Biodiversity and Conservation*, 24, 447−465.

Lemieux, J.P. & Lindgren, B.S. (2004) Ground beetle responses to patch retention harvesting in high elevation forests of British Columbia. *Ecography*, 27, 557−566.

Magura, T. (2002) Carabids and forest edge: spatial pattern and edge effect. *Forest Ecology and Management*, 157, 23−37.

Magura, T. & Tóthmérész, B. (1997)Testing edge effect on carabid assemblages in an oak-hornbeam forest. *Acta Zoologica Academiae Scientiarum Hungaricae*, 43, 303−312.

Magura, T. & Tóthmérész, B. (1998) Edge effect on carabids in an oak-hornbeam forest at the Aggtelek National Park (Hungary). *Acta Phytopathologica et Entomologica Hungarica*, 33, 379−387.

Magura, T., Tóthmérész, B. & Bordán, Zs. (2002) Carabids in an oak-hornbeam forest: testing the edge effect hypothesis. *Acta Biologica Debrecina*, 24, 55−72.

Magura, T., Tóthmérész, B. & Molnár, T. (2000) Spatial distribution of carabids along grass-forest transects. *Acta Zoologica Academiae Scientiarum Hungaricae*, 46, 1−17.

Magura, T., Tóthmérész, B. & Molnár, T. (2001) Forest edge and diversity: carabids along forest-grassland transects. *Biodiversity and Conservation*, 10, 287−300.

Máthé, I. (2006) Forest edge and carabid diversity in a Carpathian beech forest. *Community Ecology*, 7, 91−97.

Molnár, T., Magura, T., Tóthmérész, B. & Elek, Z. (2001) Ground beetles (Carabidae) and edge effect in oak-hornbeam forest and grassland transects. *European Journal of Soil Biology*, 37, 297−300.

Phillips, I.D., Cobb, T.P., Spence, J.R. & Brigham, R.M. (2006) Salvage logging, edge effects, and carabid beetles: Connections to conservation and sustainable forest management. *Environmental Entomology*, 35, 950−957.

Roume, A., Ouin, A., Raison, L. & Deconchat, M. (2011) Abundance and species richness of overwintering ground beetles (Coleoptera: Carabidae) are higher in the edge than in the centre of a woodlot. *European Journal of Entomology*, 108, 615−622.

Silverman, B., Horn, D.J., Purrington, F.F. & Gandhi, K.J.K. (2008) Oil pipeline corridor through an intact forest alters ground beetle (Coleoptera: Carabidae) assemblages in Southeastern Ohio. *Environmental Entomology*, 37, 725−733.

Sklodowski, J. (1999) Movement of selected carabid species (Col. Carabidae) through a pine forest-fallow ecotone. *Folia Forestalia Polonica*, 41, 5−23.

Soga, M., Kanno, N., Yamaura, Y. & Koike, S. (2013) Patch size determines the strength of edge effects on carabid beetle assemblages in urban remnant forests. *Journal of Insect Conservation*, 17, 421−428.

Spence, J.R., Langor, D.W., Niemelä, J., Cárcamo, H.A. & Currie, C.R. (1996) Northern forestry and carabids: the case for concern about old-growth species. *Annales Zoologici Fennici*, 33, 173−184.

Strong, A.M., Dickert, C.A. & Bell, R.T. (2002) Ski trail effects on a beetle (Coleoptera: Carabidae, Elateridae) community in Vermont. *Journal of Insect Conservation*, 6, 149−159.

Taboada, A., Kotze, D.J. & Salgado, J.M. (2004) Carabid beetle occurrence at the edges of oak and beech forests in NW Spain. *European Journal of Entomology*, 101, 555−563.

Tóthmérész, B., Nagy, D.D., Mizser, S., Bogyó, D. & Magura, T. (2014) Edge effects on ground-dwelling beetles (Carabidae and Staphylinidae) in oak forest-forest edge-grassland habitats in Hungary. *European Journal of Entomology*, 111, 686−691.

Ulyshen, M.D., Hanula, J.L., Scott, H., Kilgo, J.C. & Moorman, C.E. (2006) The response of ground beetles (Coleoptera: Carabidae) to selection cutting in a South Carolina bottomland hardwood forest. *Biodiversity and Conservation*, 15, 261−274.

Yu, X-D., Lou, T-H., Zhou, H-Z. & Yang, J. (2007) Distribution of carabid beetles (Coleoptera: Carabidae) across a forest-grassland ecotone in Southwestern China. *Environmental Entomology*, 36, 348−355.

Yu, X-D., Lou, T-H. & Zhou, H-Z. (2009) Distribution of carabid beetles (Coleoptera: Carabidae) across ecotones between regenerating and mature forests in Southwestern China. *Environmental Entomology*, 38, 1053−1060.

Yu, X-D., Lou, T-H. & Zhou, H-Z. (2010) Distribution of ground-dwelling beetle assemblages (Coleoptera) across ecotones between natural oak forests and mature pine plantations in North China. *Journal of Insect Conservation*, 14, 617−626.

**Appendix S2.** Estimates and heterogeneities in the models.

**Abundance**

| **Subgroup** | **Mean effect size (model)** | **Lower CI bound (model)** | **Upper CI bound (model)** | **SE (model)** | ***p* value (model)** | **Q (hetero-geneity)** | ***p* value (hetero-geneity)** | **Tau^2^** | **I^2^** | **R^2^** |
| --- | --- | --- | --- | --- | --- | --- | --- | --- | --- | --- |
| Edges with human influences | 0.174 | -0.283 | 0.630 | 0.233 | 0.456 | 106.219 | <0.001 | 1.819 | 79% |  |
| Edges with natural processes | 0.042 | -0.506 | 0.590 | 0.280 | 0.881 | 89.248 | <0.001 | 0.738 | 87% |  |
| Overall | 0.120 | -0.231 | 0.47 | 0.179 | 0.504 | 195.556 | <0.001 | 1.208 | 82% | 0.00% |
|  |  |  |  |  |  |  |  |  |  |  |
| Edges disturbed by agriculture | 0.102 | -0.841 | 1.044 | 0.481 | 0.833 | 41.312 | <0.001 | 2.436 | 83% |  |
| Edges disturbed by forestry | 0.166 | -0.675 | 1.007 | 0.429 | 0.699 | 47.035 | <0.001 | 3.108 | 81% |  |
| Overall | 0.137 | -0.490 | 0.765 | 0.320 | 0.668 | 88.458 | <0.001 | 2.799 | 81% | 0.00% |

| **Component of variance** | ***Q*** | ***d.f.*** | ***p*** |  | **Component of variance** | ***Q*** | ***d.f.*** | ***p*** |
| --- | --- | --- | --- | --- | --- | --- | --- | --- |
| Edges with human influences | 106.219 | 22 | <0.001 |  | Edges disturbed by agriculture | 41.312 | 7 | <0.001 |
| Edges with natural processes | 89.248 | 12 | <0.001 |  | Edges disturbed by forestry | 47.035 | 9 | <0.001 |
| Within | 195.467 | 34 | <0.001 |  | Within | 88.347 | 16 | <0.001 |
| Between | 0.131 | 1 | 0.717 |  | Between | 0.01 | 1 | 0.92 |
| Total | 195.556 | 35 | <0.001 |  | Total | 88.458 | 17 | <0.001 |

**Species richness**

| **Subgroup** | **Mean effect size (model)** | **Lower CI bound (model)** | **Upper CI bound (model)** | **SE (model)** | ***p* value (model)** | **Q (hetero-geneity)** | ***p* value (hetero-geneity)** | **Tau^2^** | **I^2^** | **R^2^** |
| --- | --- | --- | --- | --- | --- | --- | --- | --- | --- | --- |
| Edges with human influences | -0.137 | -0.479 | 0.205 | 0.174 | 0.432 | 107.077 | <0.001 | 0.8740 | 73% |  |
| Edges with natural processes | -1.474 | -1.956 | -0.992 | 0.246 | <0.001 | 52.574 | <0.001 | 0.834 | 77% |  |
| Overall | -0.584 | -0.863 | -0.305 | 0.142 | <0.001 | 224.828 | <0.001 | 0.860 | 81% | 37.16% |
|  |  |  |  |  |  |  |  |  |  |  |
| Edges disturbed by agriculture | -0.364 | -0.973 | 0.246 | 0.311 | 0.242 | 72.826 | <0.001 | 2.798 | 84% |  |
| Edges disturbed by forestry | -0.003 | -0.592 | 0.587 | 0.301 | 0.993 | 15.466 | 0.116 | 0.051 | 35% |  |
| Overall | -0.177 | -0.601 | 0.246 | 0.216 | 0.412 | 88.448 | <0.001 | 1.099 | 74% | 0.00% |

| **Component of variance** | ***Q*** | ***d.f.*** | ***p*** |  | **Component of variance** | ***Q*** | ***d.f.*** | ***p*** |
| --- | --- | --- | --- | --- | --- | --- | --- | --- |
| Edges with human influences | 107.077 | 29 | <0.001 |  | Edges disturbed by agriculture | 72.826 | 12 | <0.001 |
| Edges with natural processes | 52.574 | 12 | <0.001 |  | Edges disturbed by forestry | 15.466 | 10 | 0.116 |
| Within | 159.651 | 41 | <0.001 |  | Within | 88.291 | 22 | <0.001 |
| Between | 19.636 | 1 | <0.001 |  | Between | 0.696 | 1 | 0.404 |
| Total | 224.828 | 42 | <0.001 |  | Total | 88.448 | 23 | <0.001 |

**Abundance of forest species**

| **Subgroup** | **Mean effect size (model)** | **Lower CI bound (model)** | **Upper CI bound (model)** | **SE (model)** | ***p* value (model)** | **Q (hetero-geneity)** | ***p* value (hetero-geneity)** | **Tau^2^** | **I^2^** | **R^2^** |
| --- | --- | --- | --- | --- | --- | --- | --- | --- | --- | --- |
| Edges with human influences | 0.380 | 0.168 | 0.592 | 0.108 | 0.000 | 194.428 | <0.001 | 0.454 | 64% |  |
| Edges with natural processes | -0.048 | -0.170 | 0.075 | 0.062 | 0.444 | 789.077 | <0.001 | 0.491 | 82% |  |
| Overall | 0.059 | -0.047 | 0.165 | 0.054 | 0.274 | 1008.142 | <0.001 | 0.485 | 79% | 4.14% |
|  |  |  |  |  |  |  |  |  |  |  |
| Edges disturbed by agriculture | 0.713 | 0.275 | 1.151 | 0.223 | 0.001 | 33.044 | 0.016 | 0.3750 | 46% |  |
| Edges disturbed by forestry | 0.134 | -0.135 | 0.403 | 0.137 | 0.329 | 112.031 | <0.001 | 0.292 | 63% |  |
| Edges disturbed by urbanization | 1.233 | 0.487 | 1.978 | 0.381 | 0.001 | 23.368 | 0.001 | 2.543 | 74% |  |
| Overall | 0.374 | 0.155 | 0.594 | 0.112 | 0.001 | 185.320 | <0.001 | 0.460 | 63% | 0.00% |

| **Component of variance** | ***Q*** | ***d.f.*** | ***p*** |  | **Component of variance** | ***Q*** | ***d.f.*** | ***p*** |
| --- | --- | --- | --- | --- | --- | --- | --- | --- |
| Edges with human influences | 194.428 | 70 | <0.001 |  | Edges disturbed by agriculture | 33.044 | 18 | 0.016 |
| Edges with natural processes | 789.077 | 145 | <0.001 |  | Edges disturbed by forestry | 112.031 | 42 | <0.001 |
| Within | 983.506 | 215 | <0.001 |  | Edges disturbed by urbanization | 23.368 | 6 | 0.001 |
| Between | 11.733 | 1 | 0.001 |  | Within | 168.443 | 66 | <0.001 |
| Total | 1008.142 | 216 | <0.001 |  | Between | 10.439 | 2 | 0.005 |
|  |  |  |  |  | Total | 185.320 | 68 | <0.001 |

**Abundance of generalist species**

| **Subgroup** | **Mean effect size (model)** | **Lower CI bound (model)** | **Upper CI bound (model)** | **SE (model)** | ***p* value (model)** | **Q (hetero-geneity)** | ***p* value (hetero-geneity)** | **Tau^2^** | **I^2^** | **R^2^** |
| --- | --- | --- | --- | --- | --- | --- | --- | --- | --- | --- |
| Edges with human influences | -0.365 | -0.541 | -0.190 | 0.090 | <0.001 | 111.274 | <0.001 | 0.186 | 49% |  |
| Edges with natural processes | -0.392 | -0.497 | -0.286 | 0.054 | <0.001 | 272.733 | <0.001 | 0.189 | 65% |  |
| Overall | -0.385 | -0.475 | -0.294 | 0.046 | <0.001 | 385.281 | <0.001 | 0.188 | 60% | 0.00% |
|  |  |  |  |  |  |  |  |  |  |  |
| Edges disturbed by agriculture | -0.593 | -0.918 | -0.268 | 0.166 | 0.000 | 48.291 | 0.002 | 0.303 | 50% |  |
| Edges disturbed by forestry | -0.225 | -0.479 | 0.029 | 0.130 | 0.083 | 54.517 | 0.001 | 0.180 | 50% |  |
| Overall | -0.365 | -0.565 | -0.164 | 0.102 | <0.001 | 108.718 | <0.001 | 0.202 | 52% | 11.85% |

| **Component of variance** | ***Q*** | ***d.f.*** | ***p*** |  | **Component of variance** | ***Q*** | ***d.f.*** | ***p*** |
| --- | --- | --- | --- | --- | --- | --- | --- | --- |
| Edges with human influences | 111.274 | 57 | <0.001 |  | Edges disturbed by agriculture | 48.291 | 24 | 0.002 |
| Edges with natural processes | 272.733 | 96 | <0.001 |  | Edges disturbed by forestry | 54.517 | 27 | 0.001 |
| Within | 384.007 | 153 | <0.001 |  | Within | 102.808 | 51 | <0.001 |
| Between | 0.064 | 1 | 0.800 |  | Between | 3.061 | 1 | 0.080 |
| Total | 385.281 | 154 | <0.001 |  | Total | 108.718 | 52 | <0.001 |

**Abundance of open-habitat species**

| **Subgroup** | **Mean effect size (model)** | **Lower CI bound (model)** | **Upper CI bound (model)** | **SE (model)** | ***p* value (model)** | **Q (hetero-geneity)** | ***p* value (hetero-geneity)** | **Tau^2^** | **I^2^** | **R^2^** |
| --- | --- | --- | --- | --- | --- | --- | --- | --- | --- | --- |
| Edges with human influences | -0.241 | -0.438 | -0.044 | 0.100 | 0.017 | 34.688 | 0.119 | 0.072 | 25% |  |
| Edges with natural processes | -0.333 | -0.433 | -0.232 | 0.051 | <0.001 | 67.174 | 0.168 | 0.031 | 15% |  |
| Overall | -0.314 | -0.403 | -0.224 | 0.046 | <0.001 | 102.651 | 0.082 | 0.035 | 18% | 0.00% |
|  |  |  |  |  |  |  |  |  |  |  |
| Edges disturbed by forestry | -0.199 | -0.430 | 0.032 | 0.118 | 0.091 | 29.958 | 0.093 | 0.081 | 30% |  |
| Edges disturbed by other human influence | -0.585 | -1.134 | -0.036 | 0.280 | 0.037 | 2.511 | 0.643 | 0 | 0% |  |
| Overall | -0.257 | -0.470 | -0.044 | 0.109 | 0.018 | 34.688 | 0.119 | 0.059 | 25% | 18.12% |

| **Component of variance** | ***Q*** | ***d.f.*** | ***p*** |  | **Component of variance** | ***Q*** | ***d.f.*** | ***p*** |
| --- | --- | --- | --- | --- | --- | --- | --- | --- |
| Edges with human influences | 34.688 | 26 | 0.119 |  | Edges disturbed by forestry | 29.958 | 21 | 0.093 |
| Edges with natural processes | 67.174 | 57 | 0.168 |  | Edges disturbed by other human influence | 2.511 | 4 | 0.643 |
| Within | 101.862 | 83 | 0.078 |  | Within | 32.469 | 25 | 0.145 |
| Between | 0.664 | 1 | 0.415 |  | Between | 1.609 | 1 | 0.205 |
| Total | 102.651 | 84 | 0.082 |  | Total | 34.688 | 26 | 0.119 |

**Appendix S3.** Funnel plots of the tested variables with missing studies (empty circles), regression tests for funnel plot asymmetry, and model results after trim and fill.

**Abundance**


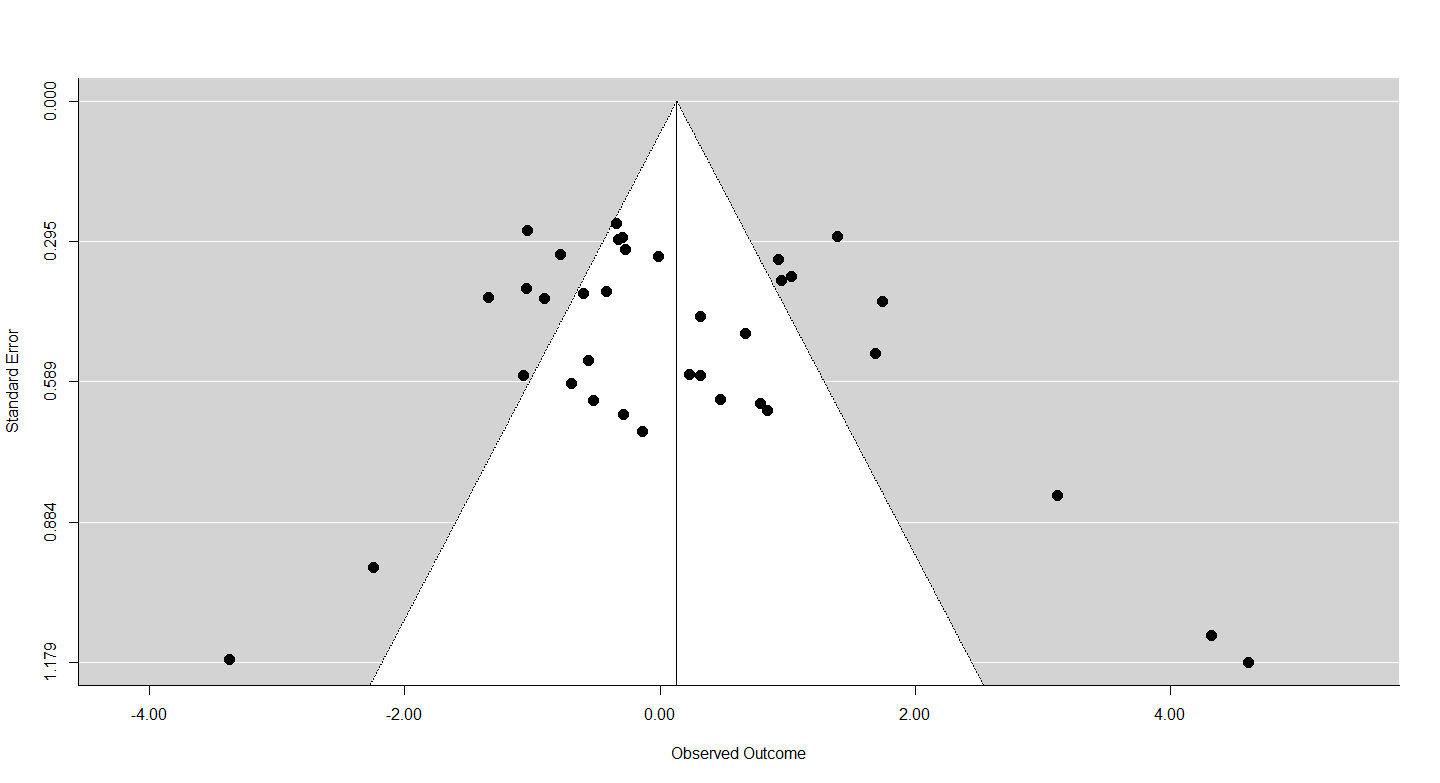


***Regression Test for Funnel Plot Asymmetry***

model: weighted regression with multiplicative dispersion

predictor: standard error

test for funnel plot asymmetry: t = 1.1749, d.f. = 34, *p* = 0.2482

model: mixed-effects meta-regression model

predictor: standard error

test for funnel plot asymmetry: z = 1.6010, *p* = 0.1094

**Species richness**


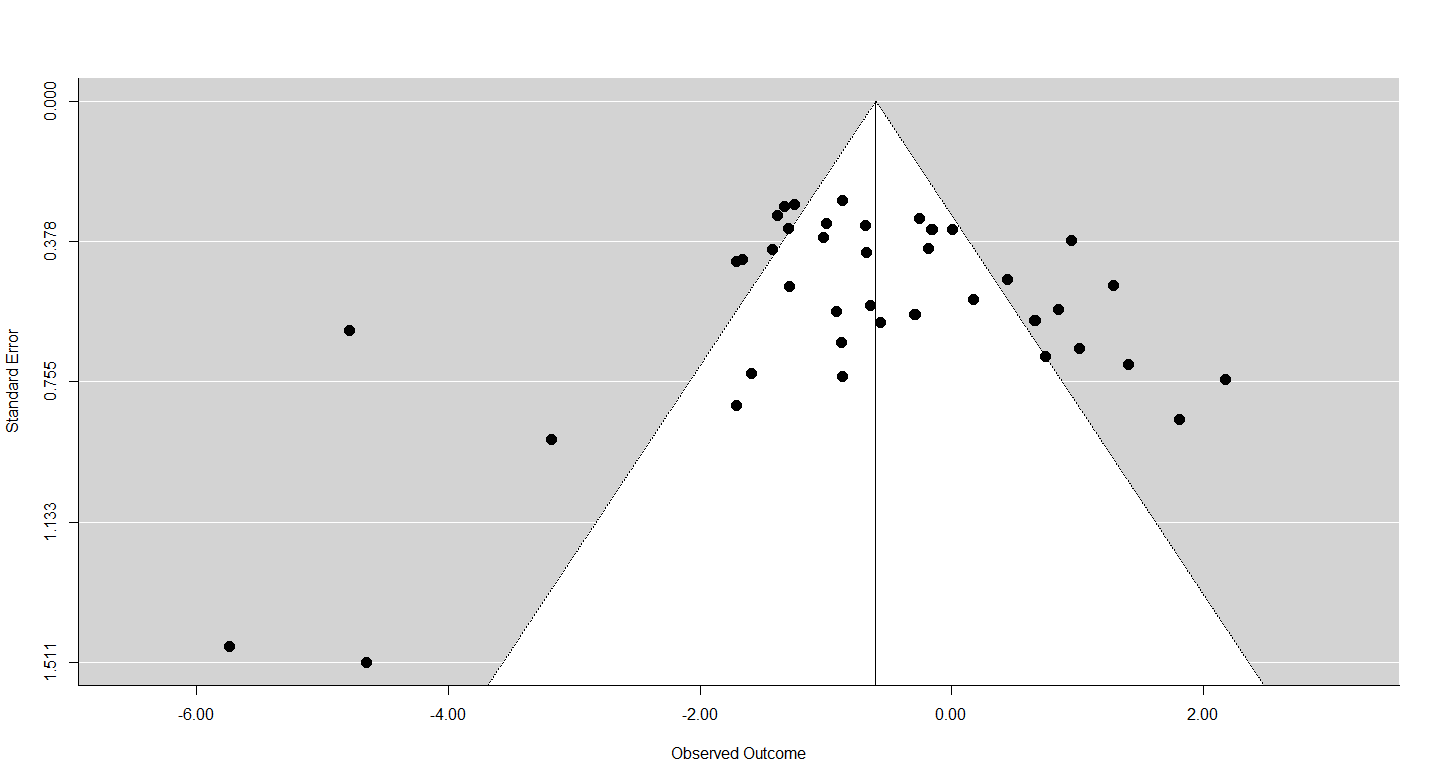


***Regression Test for Funnel Plot Asymmetry***

model: weighted regression with multiplicative dispersion

predictor: standard error

test for funnel plot asymmetry: t = 0.4178, d.f. = 41, *p* = 0.6783

model: mixed-effects meta-regression model

predictor: standard error

test for funnel plot asymmetry: z = -1.6788, *p* = 0.0932

**Abundance of forest species**


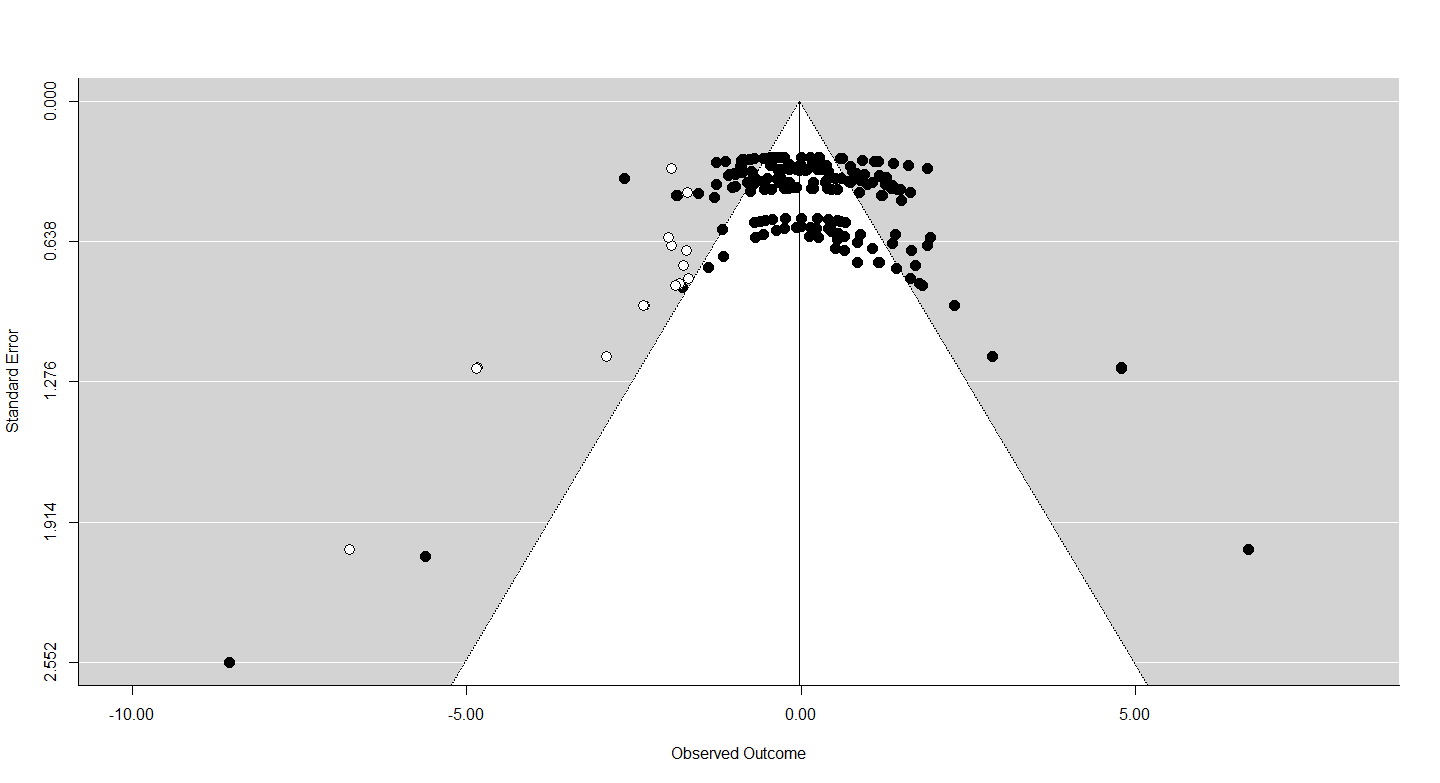


***Regression Test for Funnel Plot Asymmetry***

model: weighted regression with multiplicative dispersion

predictor: standard error

test for funnel plot asymmetry: t = 3.3411, d.f. = 215, *p* = 0.0010

model: mixed-effects meta-regression model

predictor: standard error

test for funnel plot asymmetry: z = 4.3506, *p* < .0001

***Model Results after Trim and Fill***:

Estimated number of missing studies on the left side: 15 (SE = 9.2278)

Estimate Hedge's *g* Lower bound Upper bound SE *p* value

-0.0282 -0.1461 0.0896 0.0601 0.6385

**Abundance of generalist species**


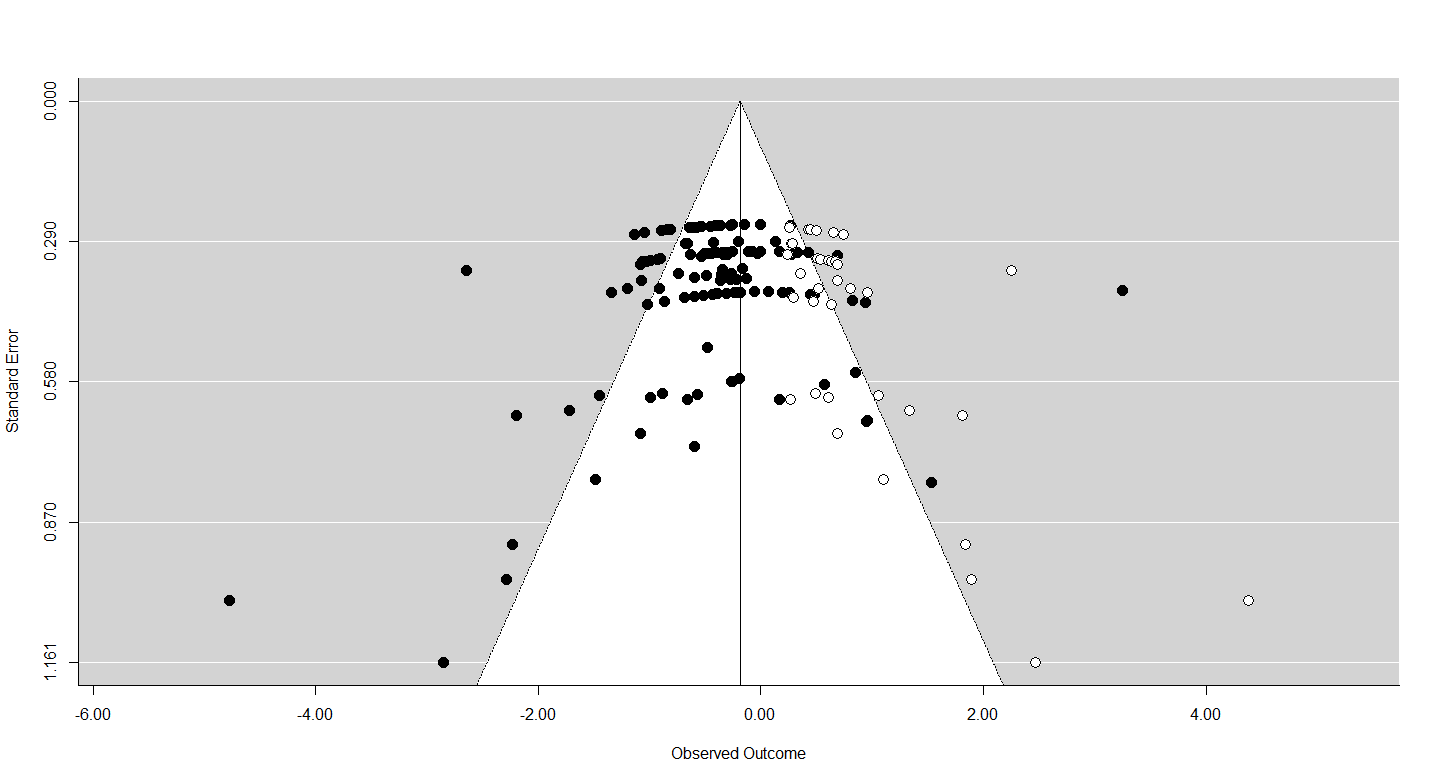


***Regression Test for Funnel Plot Asymmetry***

model: weighted regression with multiplicative dispersion

predictor: standard error

test for funnel plot asymmetry: t = -1.1038, d.f. = 153, *p* = 0.2714

model: mixed-effects meta-regression model

predictor: standard error

test for funnel plot asymmetry: z = -2.2795, *p* = 0.0226

***Model Results after Trim and Fill***:

Estimated number of missing studies on the right side: 38 (SE = 8.1196)

Estimate Hedge's *g* Lower bound Upper bound SE *p* value

-0.1875 -0.2870 -0.0881 0.0507 0.0002

**Abundance of open-habitat species**


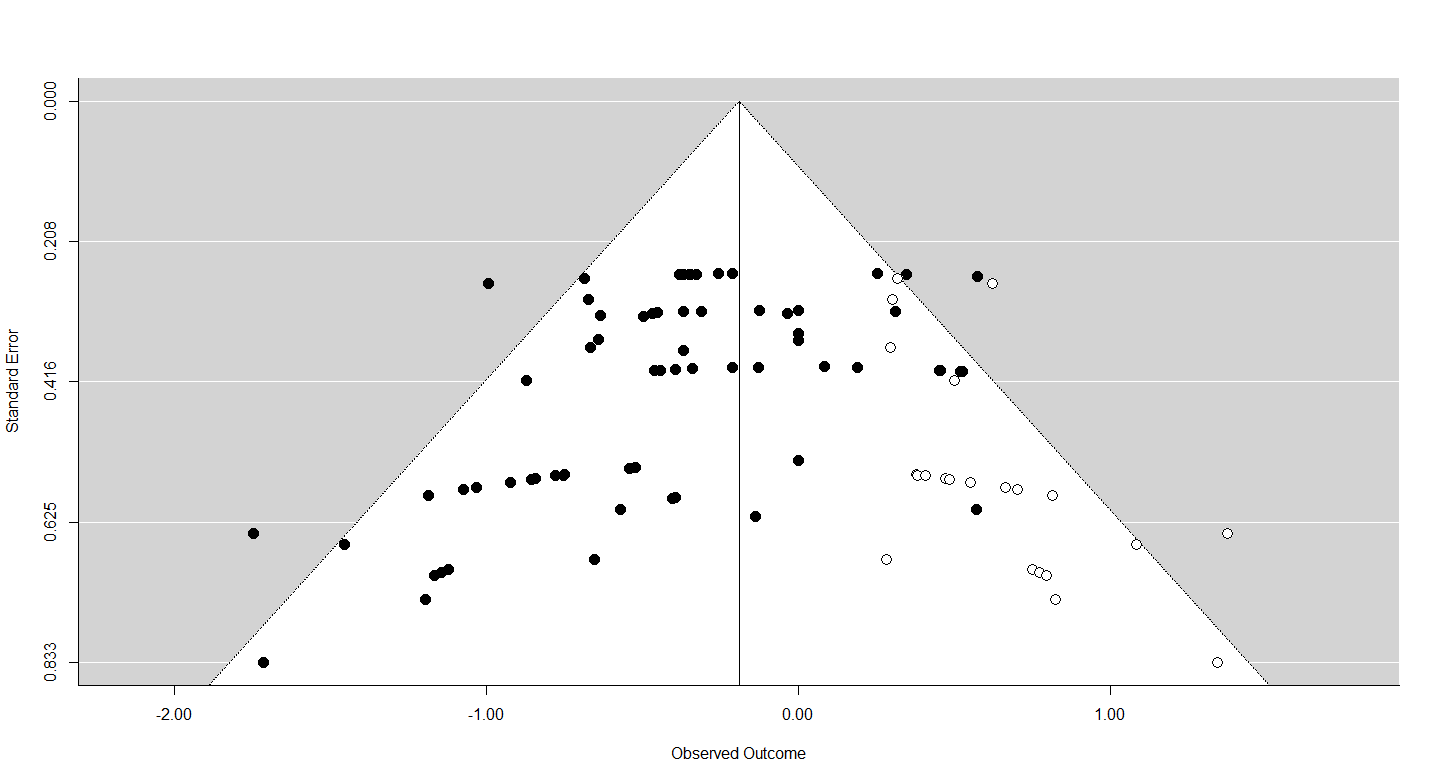


***Regression Test for Funnel Plot Asymmetry***

model: weighted regression with multiplicative dispersion

predictor: standard error

test for funnel plot asymmetry: t = -3.9390, d.f. = 83, *p* = 0.0002

model: mixed-effects meta-regression model

predictor: standard error

test for funnel plot asymmetry: z = -3.8920, *p* < .0001

***Model Results after Trim and Fill***:

Estimated number of missing studies on the right side: 23 (SE = 5.9897)

Estimate Hedge's *g* Lower bound Upper bound SE *p* value

-0.1896 -0.2826 -0.0966 0.0474 <.0001
